# A Strange Isolation Hospital

**Published:** 1921-01-29

**Authors:** 


### A Strange Isolation Hospital.

It is to be hoped that the isolation hospital of the Bishop Auckland Urban District Council is an isolated case, for Dr. Cameron, the Deputy County Medical Officer of Health for Durham, condemns the institution as quite inadequate for the requirements of the district, summarising the main defects as follows :—

(1) There is no provision for the separation of the sexes; (2) the sanitary conveniences and bathing facilities are quite inadequate; (3) the accommodation for patients and staff is also inadequate; (4) there is no provision for cases suffering from "mixed infections" or for the isolation of doubtful cases; (5) an adequate nursing and domestic staff is not maintained; (6) no proper precautions are taken to prevent patients admitted with one infectious disease contracting another.

In the rural district of Auckland, which surrounds the urban district, there are two modern, up-to-date isolation hospitals, and it is urged that the Urban District Council should arrange to make use of these and shut up its own unsuitable establishment.

